# Acute Kidney Injury Induces Lung Damage via Mitochondrial DAMPs by Activating TREM-1 and cGAS-STING Pathways

**DOI:** 10.3390/cells14211716

**Published:** 2025-10-31

**Authors:** Zhi Tian, Runze Ni, Nadezhda N. Zheleznova, Diane Allen-Gipson, Lei Wang, Vijay Subramanian, Kiran Dhanireddy, Sarah Y. Yuan, Nohely Hernandez Soto, Jose D. Herazo-Maya, Kristof Williams, Isabella Lozonschi, Andrew Bedard, Gabrielle Morrison, Ruisheng Liu

**Affiliations:** 1 Department of Molecular Pharmacology & Physiology, Morsani College of Medicine, University of South Florida (USF), 560 Channelside Dr., Tampa, FL 33602, USAnohelyh@usf.edu (N.H.S.); gabriellemorrison@usf.edu (G.M.); ruisheng@usf.edu (R.L.); 2 USF-TGH Transplant Research Center (UTRC), 560 Channelside Dr., Tampa, FL 33602, USA; 3 Department of Pharmaceutical Sciences, USF Health Taneja College of Pharmacy, University of South Florida (USF), 560 Channelside Dr., Tampa, FL 33602, USA; 4 Transplant Institute, Tampa General Hospital (TGH), 1 Tampa General Circle, Tampa, FL 33606, USA; vsubramanian@tgh.org (V.S.);; 5 Ubben Center for Pulmonary Fibrosis Research, Morsani College of Medicine, University of South Florida (USF), 560 Channelside Dr., Tampa, FL 33602, USA

**Keywords:** acute kidney injury, acute lung injury, TREM-1, cGAS-STING signaling pathway, mtDAMPs

## Abstract

Acute kidney injury (AKI) is a leading cause of distant organ dysfunction among critically ill patients. Mitochondrial dysfunction is considered a key factor driving the damage after renal ischemia–reperfusion (IR) injury. Damaged mitochondria release mitochondrial damage-associated molecular patterns (mtDAMPs) into the cytosol, which initiate a systemic inflammatory response. To better understand the underlying mechanism, mice were challenged with 30 min of bilateral renal ischemia followed by 24 h of reperfusion. The cytokine profiling in mouse lung tissues revealed that TREM-1 was significantly increased. Western Blot (WB) analysis demonstrated that the cGAS and STING pathway was increased in AKI mice. Transmission electron microscopy (TEM) images indicated that the mtDAMPs were released from damaged kidney mitochondria. Injection of mtDAMPs into mice induced an inflammatory response in the lungs similar to that induced by AKI. Mouse macrophages and lung epithelial cells were utilized to verify if inhibition of the TREM-1 and cGAS-STING pathways reduces mtDAMP-induced lung injury. Electric Cell-substrate Impedance Sensing (ECIS) results demonstrated that inhibiting the TREM-1 and cGAS-STING pathways significantly increased cell proliferation and migration while reducing mtDAMP-induced cytotoxicity. In conclusion, our findings suggest that targeting TREM-1 and cGAS-STING has the potential to attenuate acute lung injury in IR-AKI.

## 1. Introduction

Patients with acute kidney injury (AKI) have a 1.4- to 15.4-fold increase in the risk of mortality [1,2,3,4]. In addition, AKI can induce injuries beyond the kidney, leading to multi-organ dysfunction [5,6,7,8]. Among the organs affected, the lung is particularly vulnerable [5,6,7]. Acute lung injury (ALI) often accompanies AKI in critically ill patients, contributing to 80% of mortality [9], which has been demonstrated in both clinical and experimental studies.

Data from patients and animal models suggest that the pathophysiological mechanisms are complex and involve multiple pathways, but these mechanisms have not been fully clarified [10,11]. ALI induced by ischemia–reperfusion (IR)-AKI is characterized by pulmonary edema and neutrophil infiltration into lung tissues and alveolar space [6,12], which have been proposed due to systemic release of inflammatory mediators from damaged kidneys. Targeting mediators such as interleukin 6 (IL-6) and tumor necrosis factor -alpha (TNF-α) have ameliorated lung injury in animal models [7,12]; however, these approaches have not yet proven beneficial in human studies. Investigating the mechanisms of IR-AKI-induced ALI is essential to identify efficient therapeutic targets.

The pathophysiology of AKI is largely driven by cellular damage, inflammation, and oxidative stress within the kidney parenchyma [10], but systemic factors are increasingly recognized as contributing to the propagation of injury in distant organs [13]. Mitochondrial dysfunction is considered a key mechanism driving the damage after renal IR injury [14]. Damaged mitochondria release mitochondrial damage-associated molecular patterns (mtDAMPs) into the cytosol, initiating an immune response and successive pro-inflammatory events. Triggering receptor expressed on myeloid cells-1 (TREM-1) recognizes DAMPs and plays an important role in immune defense; it is known to amplify the inflammatory signals by promoting the production of pro-inflammatory mediators [15,16]. A new multi-center prospective clinical study identified TREM-1 as a good indicator for the diagnosis of sepsis-associated AKI in patients [17]; however, no research has been conducted regarding the function of TREM-1 in AKI-induced lung injury. The cyclic GMP-AMP synthase (cGAS)—stimulator of interferon genes (STING) pathway is also known to sense cytoplasmic double-stranded DNA, such as mitochondrial DNA (mtDNA), which is one of the key components of mtDAMPs. There are studies demonstrating that the cGAS-STING pathway is involved in not only cisplatin-induced, but also sepsis-associated and IR-induced kidney injury [18,19,20,21,22,23,24]. Therefore, we will also investigate its role in lung injury following IR-induced kidney injury.

The aim of this study is to elucidate how IR-induced release of kidney mtDAMPs contributes to ALI, provide insight into inter-organ molecular crosstalk, and identify potential therapeutic targets for the prevention of IR-AKI-induced multi-organ injury.

## 2. Materials and Methods

### 2.1. Animals and Bilateral IR-AKI Surgical Procedure

Ten- to twelve-week-old male C57BL/6 mice were purchased from Jackson Laboratories (Bar Harbor, ME, USA). The IR-AKI mouse model was generated as described previously [25,26,27]. Briefly, mice were anesthetized with 2–3% isoflurane, and a single dose of 1 mg/kg buprenorphine (Wedgewood Connect, San Jose, CA, USA) was administered via subcutaneous injection. A midline abdominal incision was made to expose the kidneys. Both renal pedicles were isolated and clamped for 30 min to induce renal ischemia, and the clamps were then removed. The body temperature was maintained at 36.8–37.2 °C throughout the surgery. After 24 h of reperfusion, the mice were euthanized for necropsy and tissue collection. All animal experiments were performed in accordance with the Guide for the Care and Use of Laboratory Animals of the National Institutes of Health and were approved by the University of South Florida Institutional Animal Care and Use Committees.

### 2.2. Glomerular Filtration Rate (GFR) Measurement

GFR was assessed in conscious mice via transdermal FITC–sinistrin clearance according to the manufacturer’s instructions. Briefly, mice were anesthetized with 3% isoflurane for a retro-orbital injection of FITC–sinistrin. A transdermal fluorescence monitor (MediBeacon GmbH, Mannheim, Germany) was attached to the mouse. The monitor continuously recorded the plasma fluorescence decay until the measurement automatically stopped. GFR was then calculated using the device’s software (MediBeacon Studio V2, Mannheim, Germany).

### 2.3. High-Performance Liquid Chromatography (HPLC) Measurement of Plasma Creatinine

Plasma creatinine concentrations were measured using HPLC as described previously, with slight modifications [28]. Briefly, 5 µL of plasma was added to 200 µL of HPLC-grade acetonitrile (MilliporeSigma, Burlington, MA, USA), vortexed immediately, and then centrifuged for 15 min at 13,000× *g* at 4 °C. The supernatant was transferred to a clean centrifuge tube and evaporated in a SpeedVac concentrator (Thermo Fisher Scientific, Waltham, MA, USA). The dried residue was reconstituted in 50 μL of the mobile phase and loaded for measurement in an Agilent 1260 infinity II system (Agilent, Santa Clara, CA, USA). The mobile phase, 5 mM sodium acetate (MilliporeSigma, Burlington, MA, USA), was freshly prepared using HPLC-grade water (Agilent, Santa Clara, CA, USA). Separation was achieved on a Raptor C18 2.7 μm 100 × 3.0 mm column (Restek, Bellefonte, PA, USA), with a flow rate of 0.3 mL/min. Detection of eluting creatinine peak occurred at 225 nm at 1.67 ± 0.02 min.

### 2.4. Lung Wet/Dry (W/D) Ratio

Pulmonary edema following IR-AKI was assessed by calculating the wet to dry (W/D) lung weight ratio. Lungs were weighed immediately after collection (wet lung). The wet lungs were dried at 60 °C for 48 h in a non-humidified incubator (IVYX Scientifc, Boston, MA, USA) and weighed again to obtain the dry weight (dry lung). The W/D ratio was calculated to evaluate the pulmonary edema as previously described [29].

### 2.5. Inflammatory Cell Infiltration in Broncho Alveolar Lavage Fluid (BALF) Using Flow Cytometry

BALF was collected as previously described [30]. Mice were anesthetized with a cocktail of xylazine and ketamine (0.1 mL/10 g). The trachea was cut open and cannulated with a 22 G catheter (EXEL, Redondo Beach, CA, USA). A total of 1.0 mL cold PBS (Gibco, Grand Island, NY, USA) was gently instilled through the catheter into the lungs and then recovered. The recovered BALF was used directly for flow cytometry analysis. Inflammatory cell infiltration in the BALF was measured as described by Hoecke et al. [31]. Briefly, samples were centrifuged at 400× *g* for 7 min. The supernatant was partially removed, leaving the cell pellet and 50 μL of supernatant in each tube. The cell pellets were blocked with 5 μL of TruStain FcX™ anti-mouse CD16/32 for 10 min. The Zombie NIR Fixable Viability Kit (BioLegend, San Diego, CA, USA) was used to stain for dead and live cells. Cells were then incubated with 3 μL each of PE/Dazzle 594 anti-mouse CD19, PE/Dazzle 594 anti-mouse CD3, PE/Cyanine7 anti-mouse CD11c, PE anti-mouse CD170 (Siglec-F), Brilliant Violet 42™ anti-mouse CD11b, APC anti-mouse I-A/I-E, and Alexa Fluor 488 anti-mouse Ly-6G for 15 min at room temperature. All antibodies were purchased from BioLegend (San Diego, CA, USA). Immune cell populations in BALF were analyzed using a Cytek Aurora Cytometer (Fremont, CA, USA).

### 2.6. Transmission Electron Microscopy (TEM)

Excised mouse kidneys were immediately placed in 2.5% Glutaraldehyde in 0.1 M cacodylate fixative buffer (Thermo Fisher Scientific, Waltham, MA, USA) and cut into 1 mm × 1 mm × 2 mm strips. The tissue strips were transferred to fresh fixative buffer and stored at 4 °C for 24 h. The tissue was then washed five times with 0.1 M sodium cacodylate buffer (Thermo Fisher Scientific, Waltham, MA, USA) and embedded in resin for imaging. Images were acquired using a JEOL 1400 transmission electron microscope (Tokyo, Japan). Percentage of damaged mitochondria was calculated using the grid-sampling method as described elsewhere [32]. Briefly, mitochondria images were captured at different magnifications. All mitochondria were counted, and damaged mitochondria were defined by swollen and swollen–elongated forms with vague, disordered, or disappeared cristae.

### 2.7. Proteome Profiling in Mice Lung Tissue Using Proteome Profiler Array

The proteome profiling of mouse lung tissue was performed using Mouse Cytokine Array Kit Panel A (Catalog#: ARY006, R&D systems, Minneapolis, MN, USA) according to the manufacturer’s instructions. Lung tissues were excised and homogenized in PBS with protease inhibitors. Protein concentrations were determined using a Pierce™ BCA Protein Assay Kit (Thermo Fisher Scientific, Waltham, MA, USA). A total of 200 μg of lysate was mixed with a Mouse Cytokine Array Panel A Detection Antibody Cocktail for one hour. The mixture was added to the blocked membrane and incubated overnight at 4 °C on a rocking shaker. The membranes were washed with washing buffer three times and incubated with Streptavidin–HRP for 30 min at room temperature. The proteins were detected by Chemi Reagent Mix (R&D systems, Minneapolis, MN, USA). Images were captured using ChemiDoc Imaging Systems (Bio-Rad, Hercules, CA, USA). Densitometric analysis was performed by measuring integrated density using Fiji ImageJ 1.0 (NIH, Bethesda, MD, USA).

### 2.8. Plasma and BALF Mitochondrial DNA Quantification

Mitochondrial DNA was quantified using a Mouse Absolute Mitochondrial DNA Copy Number Quantification Kit (RayBiotech, Peachtree Corners, GA, USA). DNA was isolated from plasma and BALF using a DNeasy Blood & Tissue Kit (Qiagen, Hilden, Germany). The signals were detected by a QuantStudio 7 Pro Real-Time PCR System (Applied Biosystems, Foster City, CA, USA).

### 2.9. Cell Culture and Treatment

The mouse lung bronchial epithelial cell line MM14.LU and the macrophage cell line AMJ2-C8 were purchased from American Type Culture Collection (ATCC, Manassas, VA, USA). All cell lines were grown in high-glucose Dulbecco’s modified Eagle’s medium (DMEM) (ATCC, Manassas, VA, USA) supplemented with 10% fetal bovine serum (FBS) (Gibco, Thermo Fisher Scientific). The culture medium for AMJ2-C8 cells was additionally supplemented with 5 mM 4-(2-hydroxyethyl)-1-piperazineethanesulfonic acid (HEPEs; Gibco, Thermo Fisher Scientific). TREM-1 receptor inhibitor, Nangibotide, and selective cGAS inhibitor, RU.521, were purchased from MedChemExpress and diluted in dimethyl sulfoxide (DMSO) (Thermo Fisher Scientific, Waltham, MA, USA) to final working concentrations of 50 μg/mL and 10 μM, respectively.

### 2.10. Preparation of mtDAMPs

Mouse kidneys were collected immediately following euthanasia. The tissues were processed using a mitochondria extraction kit with a gentleMACS Octo Dissociator (Miltenyi Biotec, Bergisch Gladbach, Germany) for mechanical dissociation. Homogenized tissue was subjected to differential centrifugation to isolate crude mitochondrial pellets. Mitochondria were then resuspended in DMEM and subjected to sonication on ice—30 s on, 30 s off—for 10 cycles to lyse mitochondria and release mtDAMPs. The sonicated samples were centrifuged at 12,000× *g* for 10 min, followed by 100,000× *g* for 30 min at 4 °C to remove membrane debris. The resulting supernatant containing mtDAMPs was carefully collected and quantified using a Pierce™ BCA Protein Assay Kit (Thermo Fisher Scientific, Waltham, MA, USA).

### 2.11. Taqman Real-Time PCR (RT-PCR)

Total RNA was extracted using TRIzol^®^ reagent (Invitrogen, Waltham, MA, USA) according to the manufacturer’s instructions. RT-PCR was conducted using a Taqman RNA-to-Ct 1-Step Kit (Applied Biosystems, Foster City, CA, USA) with the following primers: GAPDH (TaqMan™ Gene Expression Assay: Mm99999915_g1), TREM-1 (TaqMan™ Gene Expression Assay: Mm07295373_m1), cGAS (TaqMan™ Gene Expression Assay: Mm00557694_m1), STING (TaqMan™ Gene Expression Assay: Mm01158116_m1). The relative fold change was calculated by the 2−ΔΔCt method.

### 2.12. Western Blot

Total protein was isolated using TRIzol^®^ reagent (Invitrogen, Waltham, MA, USA) according to the manufacturer’s instructions. Samples were separated by 4–15% polyacrylamide gel electrophoresis, transferred to polyvinylidene difluoride (PVDF) membranes, blocked in 5% milk, and probed with primary antibodies—anti-c-GAS (1:1000, Cell signaling, Danvers, MA, USA), anti-STING (1:1000), anti-GAPDH (1:5000, Cell signaling, Danvers, MA, USA), and anti-β-actin (1:5000, Sigma, Burlington, MA, USA)—overnight at 4 °C. After washing in TBST and incubation with the secondary antibody for 1 h at room temperature, proteins were detected by enhanced chemiluminescence (Thermo Fisher Scientific, Waltham, MA, USA). Densitometric analysis was performed using ImageJ (NIH, USA).

### 2.13. Enzyme-Linked Immunosorbent Assay (ELISA)

The concentration of soluble TREM-1 in cell culture media was measured using Quantikine ELISA mouse TREM-1 kit (R&D systems, Minneapolis, MN, USA). A total of 50 μL of cell culture media was added to a 96-well ELISA plate and incubated for two hours at room temperature. After rinsing with washing buffer 5 times, the conjugate was added to each well and incubated for another two hours. The signals were developed with substrate solution in the dark for 30 min, and the absorbance was measured at 450 nm.

### 2.14. Cell Proliferation Assay

96W10idf ECIS Cultureware was used for cell proliferation assays as described previously [30,33]. MM14.Lu cells (2 × 10^3^) and AMJ2-C8 cells (0.2 × 10^3^) were seeded in each well and cultured in an incubator at 37 °C with 5% CO_2_. Nangibotide and RU.521 were added to the cells simultaneously, and mtDAMPs were added after 2 h of incubation. The resistance was recorded in real-time at 4000 Hz using an ECIS Zθ instrument (Applied BioPhysics, Troy, NY, USA).

### 2.15. Cell Migration Assay

8W1E ECIS Culturewares (Applied BioPhysics, Troy, NY, USA) were used for migration assays as described previously [30,33]. Briefly, MM14.Lu (4 × 10^4^) and AMJ2-C8 cells (0.4 × 10^4^) were seeded per well and cultured in an ECIS incubator at 37 °C with 5% CO_2_. Nangibotide and RU.521 were added to the cells simultaneously, and mtDAMPs were added after two hours of incubation. Cell monolayers were wounded using an elevated field pulse of 3000 μA at 80,000 Hz for 20 sec, producing a uniform circular lesion with a diameter of 250 μm. The wounds were tracked in real-time at 4000 Hz using an ECIS Zθ instrument (Applied BioPhysics, Troy, NY, USA).

### 2.16. Cell Cytotoxicity Assay

96W10idf ECIS Culturewares (Applied BioPhysics, Troy, NY, USA) were used for cell cytotoxicity assays as described elsewhere [34,35]. Briefly, MM14.Lu (6 × 10^3^) and AMJ2-C8 cells (0.6 × 10^3^) were seeded in each well and cultured in an ECIS incubator at 37 °C with 5% CO_2_. All stimulations and treatments were applied to each well. The resistance was recorded in real-time at 4000 Hz using an ECIS Zθ instrument (Applied BioPhysics, Troy, NY, USA).

### 2.17. Data Analysis and Statistics

All data are presented as mean ± SE. Data were statistically analyzed using GraphPad Prism Version 10.5 (GraphPad software, San Diego, CA, USA). Statistical differences between groups were determined using one-way ANOVA followed by Tukey’s multiple-comparison test. A single asterisk (*) means the result is statistically significant (*p* < 0.0332), ** indicates *p* < 0.0021, *** denotes *p* < 0.0002, **** represents *p* < 0.0001.

## 3. Results

### 3.1. IR-AKI Induces Remote Organ Lung Injury in Mice

**Figure 1:**
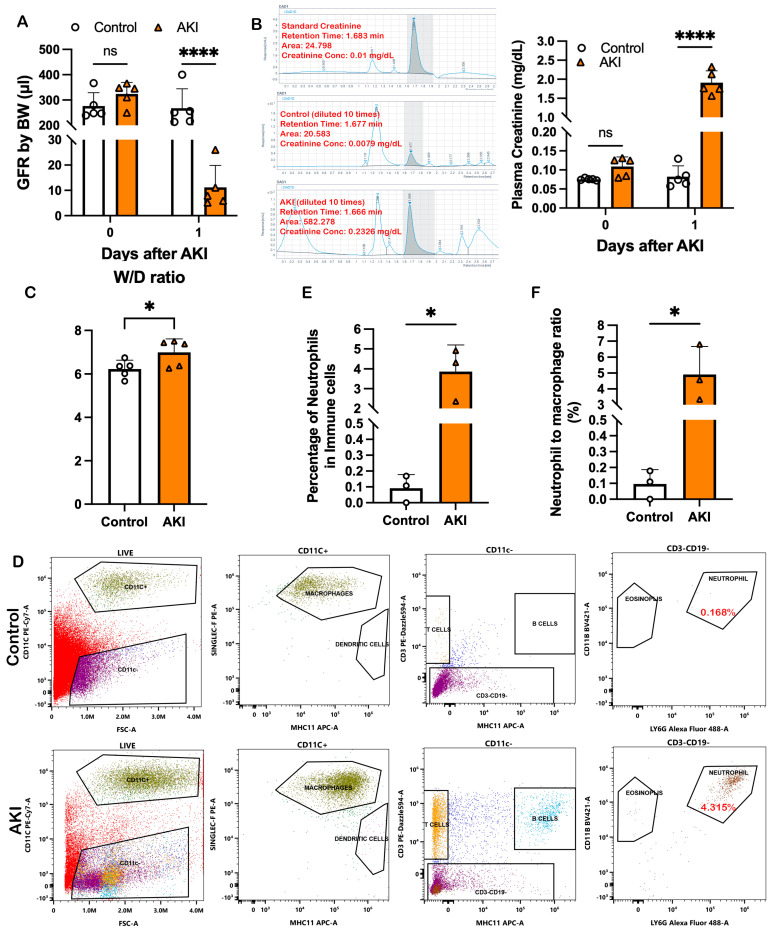
IR-AKI induces remote organ lung injury. (**A**) 30 min IR-AKI significantly reduced GFR in mice. (**B**) Mouse plasma creatinine levels measured by HPLC were dramatically elevated after IR. (**C**) Lung edema induced by IR-AKI was confirmed with wet/dry ratio. (**D**) Neutrophil infiltration in BALF was observed in IR-AKI mice using flow cytometry. (**E**) Percentage of neutrophils in immune cells. (**F**) Neutrophil to macrophage ratio in BALF. All data were analyzed using GraphPad Prism with Student’s unpaired *t*-test, *n* = 3–5 in each group, ns stands for not significant, * means *p* < 0.0332, **** represents *p* < 0.0001.

Mouse GFR and plasma creatinine levels were measured to confirm the severity of IR-AKI surgery. The GFR, normalized by body weight, saw an average reduction of 96.5%, from 323.9 μL to 11.22 μL (Figure 1A), while plasma creatinine was elevated by 20-fold (Figure 1B) after IR compared to the control group. To evaluate lung injury after IR-AKI, the lung wet/dry ratio was calculated as an indicator of pulmonary edema. The results showed that the wet/dry ratio from AKI mice was significantly increased in comparison to the control group (Figure 1C). Neutrophil infiltration in BALF is considered a key sign of acute lung damage. Our flow cytometry data further indicated that both the neutrophil counts and the neutrophil to macrophage ratio were dramatically increased in BALF isolated from IR mice (Figure 1D–F).

Collectively, these data indicate that the lungs were injured following IR-AKI surgery.

### 3.2. IR-AKI Facilitated Leakage of mtDNA

**Figure 2:**
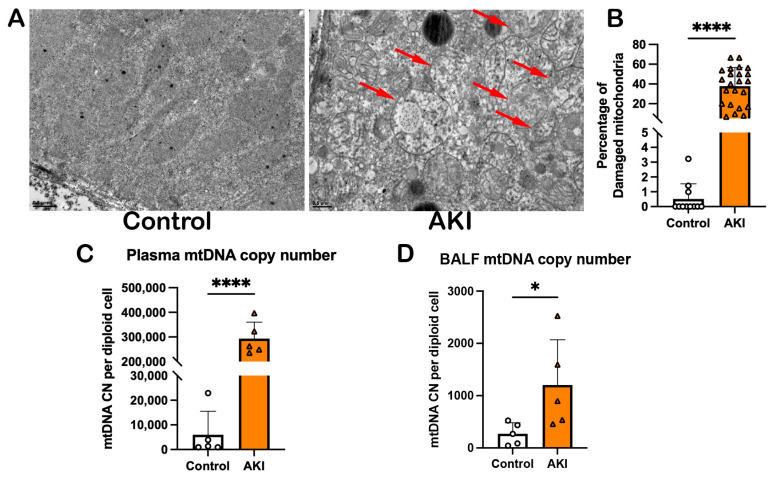
Kidney mitochondria are damaged, and mtDNA is released to lung tissue after IR. (**A**) TEM images of mitochondria in kidney tissues. Red arrows indicate damaged mitochondria. Scale bars, 0.5 μm. (**B**) Percentage of damaged mitochondria in each group. (**C**) Plasma mtDNA copy number (CN) was significantly elevated in AKI mice. (**D**) BALF mtDNA CN was increased after IR. All data were analyzed using GraphPad Prism with Student’s unpaired *t*-test, * means *p* < 0.0332, **** represents *p* < 0.0001.

Mitochondrial damage often happens after IR-AKI. To investigate the impact of renal mitochondrial dysfunction on remote organs, we first observed the effects of IR-AKI on kidney mitochondria. TEM images showed that damaged mitochondria were increased from 0.5% to 37.84% after IR-AKI, which were characterized by swollen and swollen–elongated with vague, disordered cristae (Figure 2A,B). The plasma mtDNA and BALF mtDNA also increased in IR mice compared to the control group (Figure 2C,D). The data demonstrated that IR-AKI caused kidney mitochondrial damage, and the damaged mitochondria were released to the lung tissue.

### 3.3. TREM-1 and cGAS-STING Signaling Pathways Were Activated in Lung Tissue

**Figure 3:**
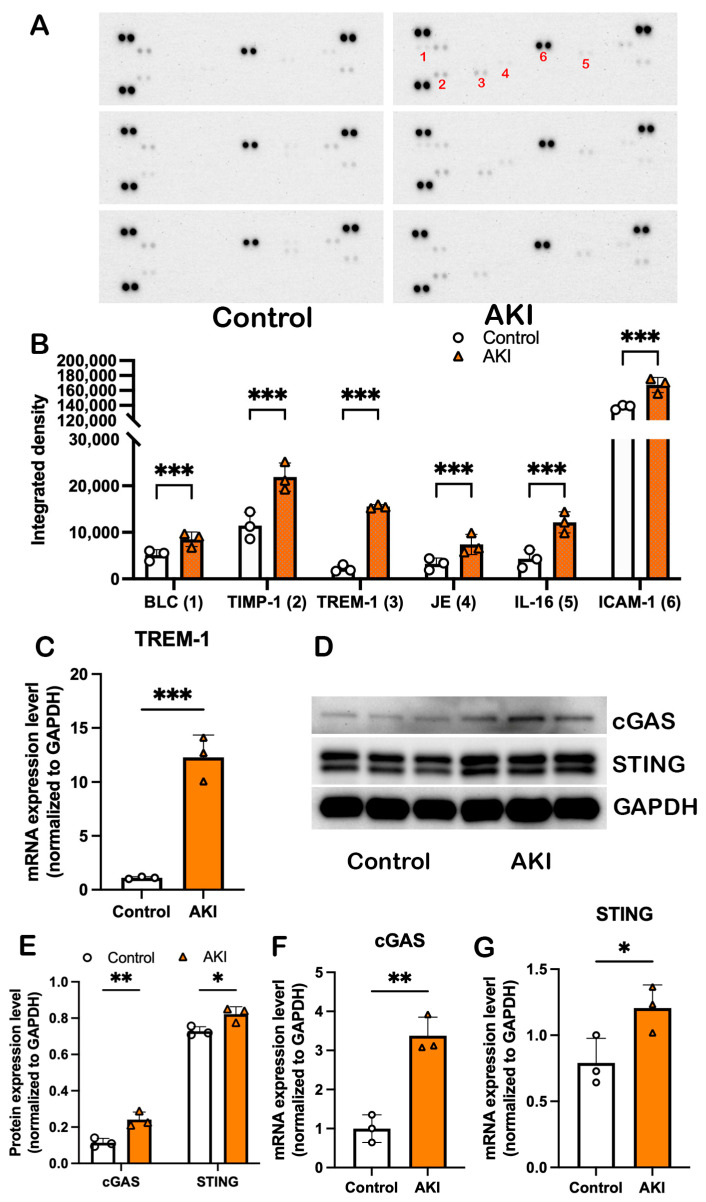
TREM-1 and cGAS-STING signaling are involved in IR-AKI-induced distant organ lung injury. (**A**) Proteome profiles of mouse lung tissue after IR-AKI; only proteins with significant changes are numbered. (**B**) Densitometry analysis of significantly changed proteins. (**C**) mRNA expression levels of TREM-1 in lung tissue. (**D**) WB showing expression levels of cGAS and STING proteins in lung tissue. (**E**) Densitometry analysis of WB results of cGAS and STING protein expression levels. (**F**) mRNA expression levels of cGAS in lung tissue. (**G**) mRNA expression levels of STING in lung tissue. Data are expressed as mean ± SE. All data are analyzed using GraphPad Prism with Student’s unpaired *t*-test, * means *p* < 0.0332, ** indicates *p* < 0.0021, *** denotes *p* < 0.0002.

To explore the molecular mechanism by which damaged mitochondria contribute to distant organ lung injury in IR-AKI mice, we first evaluated 40 mouse cytokines, chemokines, and acute phase proteins in lung tissue using a Proteome Profiler assay (Figure 3A). The results demonstrated that BLC (B lymphocyte chemoattractant), TIMP-1 (Tissue Inhibitor of Metalloproteinases-1), TREM-1, JE (CCL2/JE/MCP-1, Monocyte Chemoattractant Protein-1), IL-16 (interleukin-16), and ICAM-1 (Intercellular Adhesion Molecule 1) were significantly increased after IR, especially the expression level of TREM-1, which increased 7-fold (Figure 3B). The elevated TREM-1 production in lung tissue was confirmed by quantitative real-time PCR (Figure 3C).

TREM-1 is a pattern recognition receptor whose activation by DAMPs leads to the release of pro-inflammatory cytokines and chemokines. Damaged mitochondria release mtDAMPs, including mtDNA, and the cGAS-STING signaling pathway is known to play a crucial role in mtDNA-induced inflammation in sepsis-induced AKI [18,19]. We thus assessed the cGAS-STING expression level in the IR-AKI-induced lung tissue; both the protein expression level (Figure 3D,E) and the mRNA expression level (Figure 3F,G) of cGAS and STING were elevated after IR.

### 3.4. mtDAMPs Induce ALI Through Activation of the TREM-1 and cGAS-STING Pathway

**Figure 4:**
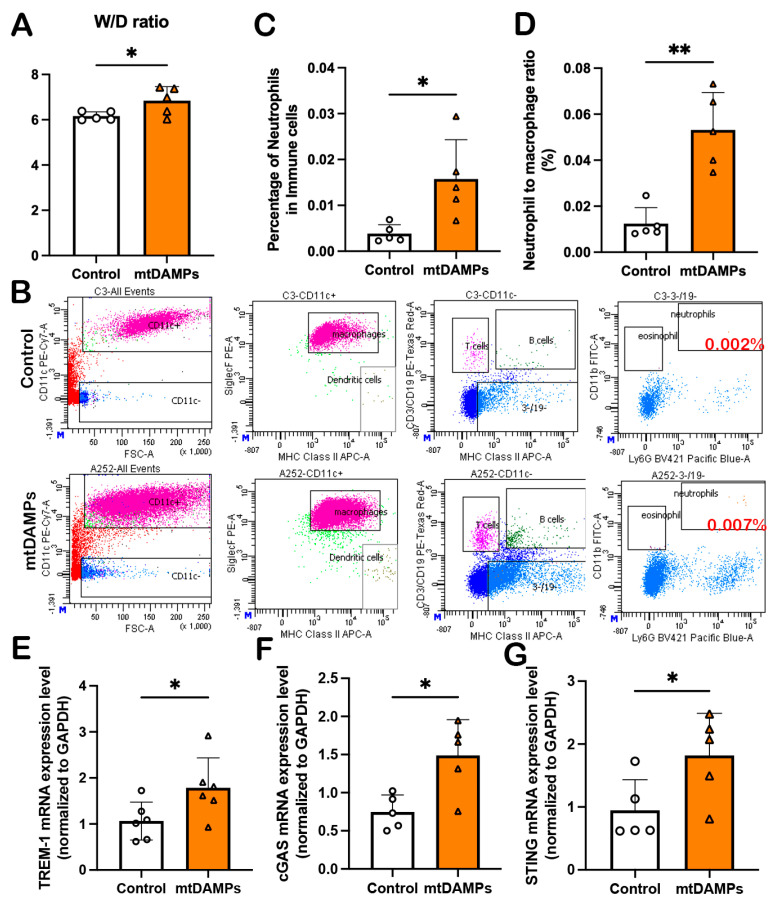
mtDAMPs induce lung injury through activation of the TREM-1 and cGAS-STING pathways. (**A**) Kidney mtDAMPs increase lung wet/dry ratio. (**B**) Neutrophil infiltration in BALF was observed in mtDAMP-treated mice using flow cytometry. (**C**) Percentage of neutrophils in immune cells. (**D**) Neutrophil to macrophage ratio in BALF. (**E**) mRNA expression levels of TREM-1 in mtDAMP-treated mouse lung tissue. (**F**) mRNA expression levels of cGAS in mtDAMP-treated mouse lung tissue. (**G**) mRNA expression levels of STING in mtDAMP-treated mouse lung tissue. All data are analyzed using GraphPad Prism with Student’s unpaired *t*-test; *n* = 5–6 in each group, and * means *p* < 0.0332, ** indicates *p* < 0.0021.

To determine whether mtDAMPs released from kidneys could induce lung injury, mice were administered 1 mL of 200 μg/mL kidney mtDAMPs via intraperitoneal injection. Mice lung samples were collected after 24 h, and the W/D ratios were increased in the mtDAMP group compared to control mice (Figure 4A). We also detected the neutrophil infiltration in BALF (Figure 4B–D), as well as overexpressed TREM-1 (Figure 4E), cGAS (Figure 4F), and STING (Figure 4G) when compared with the control group. These data imply that the mtDAMPs released from the kidneys are at least partly responsible for the distant organ lung injury during IR-AKI by activating the TREM-1 and cGAS-STING pathways.

### 3.5. mtDAMPs Increase TREM-1 Expression and Activate cGAS-STING Pathway In Vitro

**Figure 5:**
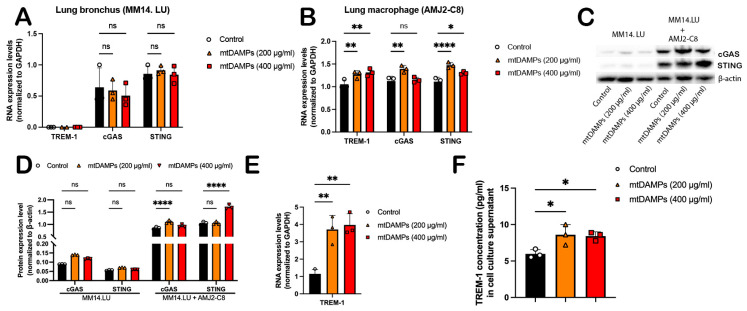
mtDAMPs increase TREM-1 expression and activate the cGAS-STING pathway in vitro. (**A**) mRNA expression levels of TREM-1, cGAS, and STING after treatment with mtDAMPs in lung bronchus cell line MM14.LU. (**B**) mRNA expression levels of TREM-1, cGAS, and STING after treatment with mtDAMPs in lung macrophage cell line AMJ2-C8. (**C**) WB results of MM14.LU cultured either alone or with AMJ2-C8 after treatment with mtDAMPs. (**D**) Densitometry analysis of WB results of MM14.LU cultured either alone or with AMJ2-C8 after treatment with mtDAMPs. (**E**) mRNA expression levels of TREM1 in AMJ2-C8 after mtDAMP treatment. (**F**) ELISA results of soluble TREM-1 protein in cell culture media after treatment with mtDAMPs. All results are presented as mean ± SE. Data were statistically analyzed using GraphPad Prism with one-way ANOVA followed by Tukey’s multiple-comparison test. ns represents statistically not significant, * means *p* < 0.0332, ** indicates *p* < 0.0021, **** represents *p* < 0.0001.

To validate the modulatory function of the TREM-1 and cGAS-STING pathways in mtDAMP-induced lung injury, mouse bronchus cells (MM14.LU) were stimulated with mtDAMPs. However, the results indicated that bronchus cells did not respond to mtDAMPs: the mRNA expressions of TREM-1, cGAS, and STING exhibited no significant differences compared to the control group (Figure 5A). We then treated lung macrophages (AMJ2-C8) with mtDAMPs; the mRNA expression levels of TREM-1, cGAS, and STING were all increased after treatment (Figure 5B), especially in the 200 μg/mL group. Therefore, we co-cultured the MM14.LU with AMJ2-C8 at a ratio of 10:1 and treated the co-cultured cells with mtDAMPs. MM14.LU is an adherent cell line, and the AMJ2-C8 is a suspension cell line that is easily separated from MM14.LU. We then removed the AMJ2-C8 cells from the cell culture dish and harvested the MM14.LU cells. The WB results demonstrated that the protein expression level of cGAS and STING in MM14.LU cells did not have any significant changes after mtDAMP treatment, consistent with the mRNA expression data; however, after co-culture with AMJ2-C8 cells, both cGAS and STING in MM14.LU were increased while treated with mtDAMPs (Figure 5C,D). Similarly, the mRNA expression of TREM-1 was elevated (Figure 5E) after treatment with mtDAMPs. The ELISA results also showed that the soluble TREM-1 protein level in the co-culture cell media was increased (Figure 5F) after treatment with mtDAMPs.

Collectively, our data suggest that kidney mtDAMPs increase TREM-1 expression and activate the cGAS-STING pathway in vitro.

### 3.6. Inhibition of Both TREM-1 and cGAS-STING Pathways Reduces mtDAMP-Induced Injuries

**Figure 6:**
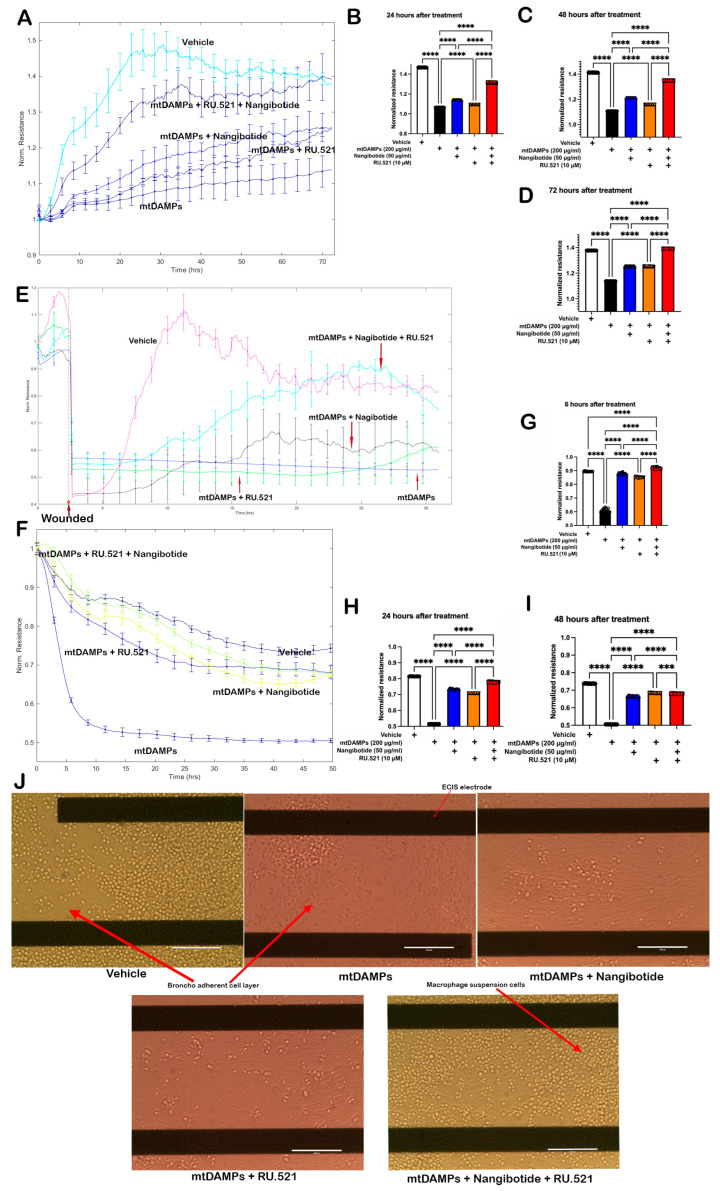
Inhibition of TREM-1 and cGAS-STING pathways reduces mtDAMP-induced injuries in vitro. (**A**) ECIS cell proliferation assay profile plots. MM14.Lu cells (2 × 10^3^) and AMJ2-C8 cells (0.2 × 10^3^) were seeded in each well. Signals were recorded in real-time at 4000 Hz. (**B**) mtDAMPs decreased cell proliferation; targeting TREM-1 and cGAS-STING ameliorates mtDAMP-impaired cell proliferation at 24 h, 48 h (**C**), and 72 h (**D**). (**E**) Targeting TREM-1 and cGAS-STING ameliorates mtDAMP-impaired cell migration. MM14.Lu cells (4 × 10^4^) and AMJ2-C8 cells (0.4 × 10^4^) were seeded in each well, and the cell monolayers were wounded using an elevated field pulse of 3000 μA at 80,000 Hz applied for 20 sec. Resistance was measured at 4000 Hz. (**F**) ECIS cell cytotoxicity assay profile plots. MM14.Lu cells (6 × 10^3^) and AMJ2-C8 cells (0.6 × 10^3^) were seeded in each well. (**G**) Cells treated with TREM-1 and cGAS inhibitors in combination were better protected against mtDAMP-induced cell toxicity during the first 6 h, 24 h (**H**), and 48 h (**I**); signals were recorded in real-time at 4000 Hz. (**J**) Cytotoxicity observed in cells treated with mtDAMPs, as characterized by fewer bronchus cells and severe damage to the confluent cell layer; treatment with TREM-1 and/or cGAS inhibitors preserved the bronchus cell layer. Data were statistically analyzed using GraphPad Prism with one-way ANOVA followed by Tukey’s multiple-comparison test. *** denotes *p* < 0.0002, **** represents *p* < 0.0001.

Since stimulating the co-culture cells with 200 μg/mL of mtDAMPs successfully upregulated TREM-1 and cGAS-STING signaling, the co-cultured cells were treated with a TREM-1 inhibitor (Nangibotide) and/or a cGAS inhibitor (RU.521), and cell behaviors were measured in real-time using an ECIS instrument. The ECIS proliferation assay (Figure 6A) showed that the mtDAMP treatment significantly decreased the cell proliferation rate, and cells treated with either Nangibotide or RU.521 ameliorated mtDAMP-induced damage. Moreover, treating cells with Nangibotide and RU.521 in combination exhibited improved protection against mtDAMPs at 24 h (Figure 6B), 48 h (Figure 6C), and 72 h (Figure 6D). Consistent with the proliferation data, the cell migration assay showed a similar trend. Once the ECIS generated a circular wound with a diameter of 250 μm in the confluent cell layer, it only took 7 h to heal in the vehicle control group, while mtDAMPs and mtDAMPs with either Nangibotide or RU.521 all failed to close the wound within 30 h. In contrast, the mtDAMPs with Nangibotide and RU.521 combination group was able to heal within 20 h after wounding (Figure 6E). Figure 6F showed that cells exposed to mtDAMPs had significantly lower resistance compared to vehicle control, and the cells treated with a combination of Nangibotide and RU.521 demonstrated better protection against mtDAMP-induced damage at 6 h (Figure 6G), 24 h (Figure 6H), and 48 h (Figure 6I). The cell images taken after the cytotoxicity assay visualized the damage caused by mtDAMPs; the mtDAMP-treated group had fewer bronchus cells, and the confluent cell layer was severely damaged compared to other groups (Figure 6J).

Taken together, mtDAMPs significantly decreased cell proliferation and migration rates and caused lung damage. Co-inhibition of both the TREM-1 and cGAS-STING pathways resulted in improved protection against the damage induced by mtDAMPs compared to inhibition of either alone.

## 4. Discussion

IR-AKI is a life-threatening disease that is associated with significantly increased risks for mortality, length of hospital stay, and healthcare-associated costs [36]. ALI is one of the most common complications in AKI patients. The underlying pathogenesis of ALI in AKI patients is still not completely understood; however, the effects of AKI on the lungs are largely due to pulmonary edema and inflammation in the initiation and progression of lung injury in clinical settings [37]. In this study, pulmonary edema and neutrophil infiltration in BALF were also observed, and several cytokines in lung tissue were dramatically elevated 24 h after 30 min of bilateral IR-AKI in the mouse model.

Our results demonstrated that among multiple pro-inflammatory factors that were upregulated in the lungs following AKI, TREM-1 increased most significantly. TREM-1 is a pattern recognition receptor expressed in macrophages and neutrophils and plays a critical role in the immune response. There is evidence demonstrating that blocking TREM-1 ameliorates lipopolysaccharide (LPS)-induced ALI [38,39]. However, to the best of our knowledge, no studies have explained its role in the case of ALI following IR-AKI, particularly in lung injury caused by damaged mitochondria.

The kidneys have the second-highest number of mitochondria after the heart, and mitochondria play a crucial role in ATP generation, reactive oxygen species generation, and antioxidant activity [40]. Mitochondrial damage is a direct consequence of IR injury. Our data also support this theory, approximately forty percent of mitochondria in the kidneys were damaged after 24 h reperfusion following 30 min IR injury. When cells are damaged, they release DAMPs; several DAMPs have been verified as activating ligands of TREM-1, such as high mobility group box 1 (HMGB1), heat shock protein 70 (HSP70), extracellular cold-inducible RNA-binding protein (eCIRP), et al. [41]. In our study, we demonstrated that TREM-1 is also activated by mtDAMPs, both in vivo and in vitro. The component of mtDAMPs is different from DAMPs from other cells; it not only includes proteins like HMGB1 and HSP70, considered TREM-1 activators, but also contains “bacterial-like” mtDNA, which is recognized by immune receptors such as cGAS.

The genetic code within mitochondria differs slightly from the host cell’s nucleus; the unmethylated CpG and formylated peptides released from mtDNA can be recognized as “foreign molecules” by the host immune system [42,43]. Thus, mtDNA has been considered the major component in the mtDAMP signaling pathways. The cGAS-STING pathway has recently garnered attention for its role in mediating immune responses to cellular stress, DNA damage, and infection [44,45]. Studies indicated that this pathway may also be activated in distant organs, such as the lung, following renal ischemia–reperfusion injury, thereby amplifying the inflammatory response and exacerbating injury [46]. cGAS is encoded by the CGAS gene and located in the cell’s nucleus, cytoplasm, and membrane [47]. It recognizes endogenous and exogenous double-stranded DNA (ds-DNA), such as mtDNA, and further synthesizes cGAMP [48]. cGAMP then binds to STING and activates the cGAS-STING signaling pathway [49]. Studies have demonstrated that the cGAS-STING axis is activated by mtDNA in cisplatin-induced and sepsis-associated kidney injury [18,19,20,21,22,23,24], and suggested that inhibiting the cGAS-STING axis may reduce the damage caused by AKI [19]. In IR-induced AKI, both Song and Feng groups observed that the release of mtDNA triggers the activation of the cGAS-STING pathway and promotes inflammation [50,51]; however, neither of them focused on lung injury in distant organs. Therefore, we decided to block both TREM-1 and cGAS-STING signaling pathways in order to minimize mtDAMP-initiated organ crosstalk at both the protein and mtDNA levels.

TREM-1 and cGAS-STING pathways are not organ-specific pathways; studies have demonstrated that targeting TREM-1 or the cGAS-STING pathway could affect the development of AKI [46,52]. Hence, we utilized mouse lung cell lines to investigate if inhibition of both signals could improve mtDAMP-induced lung injury. In our in vitro experiment, we used 200 μg/mL and 400 μg/mL of mtDAMPs to treat cells, as Zhang et al. reported that human neutrophils exposed to 400 μg/mL mtDAMPs demonstrated increased IL-8 secretion [53]. As we expected, the lung bronchus cell line MM14. LU did not react to mtDAMPs at all; however, once we treated macrophages with mtDAMPs, they sent signals to bronchus cells and affected cell behavior, such as proliferation, migration, and cytotoxicity. This indicates that the mechanisms behind mtDAMP-induced organ crosstalk are at least partially mediated by macrophages.

In this study, we demonstrated that IR-AKI damages kidney mitochondria, resulting in the release of mtDAMPs to the lung, which subsequently activates the TREM-1 and cGAS-STING pathways, ultimately leading to acute lung injury. Moreover, co-targeting the TREM-1 and cGAS-STING pathways exhibited more potent inhibitory effects on improving IR-AKI-induced ALI than targeting either individually.

## Data Availability

The original contributions presented in this study are included in the article. Further inquiries can be directed to the corresponding author.
